# Fast Determination of Ingredients in Solid Pharmaceuticals by Microwave-Enhanced In-Source Decay of Microwave Plasma Torch Mass Spectrometry

**DOI:** 10.1007/s13361-017-1708-x

**Published:** 2017-06-19

**Authors:** Rui Su, Xinchen Wang, Changming Hou, Meiling Yang, Keke Huang, Huanwen Chen

**Affiliations:** 1 Department of Chemistry, Jilin University, Changchun, 130012 China; 2 Jiangxi Key Laboratory for Mass Spectrometry and Instrumentation, East China University of Technology, Nanchang, Jiangxi 330013 China; 3 Changchun University of Chinese Medicine, Changchun, 130117 China

**Keywords:** Microwave plasma torch, Ambient ionization, Mass spectrometry, Drug screening, Mechanism, Qualitative and quantitative analysis

## Abstract

Rapid qualitative and quantitative analysis of solid samples (e.g., pharmaceutical preparations) by using a small and low-resolution mass spectrometer without MS/MS function is still a challenge in ambient pressure ionization mass spectrometric analysis. Herein, a practically efficient method termed microwave-enhanced in-source decay (MEISD) using microwave plasma torch desorption ionization coupled with time-of-flight mass spectrometry (MPTDI-TOF MS) was developed for fast analysis of pharmaceutical tablets using a miniature TOF mass spectrometer without tandem mass function. The intensity of ISD fragmentation was evaluated under different microwave power values. Several factors, including desorption distance and time that might affect the signal intensity and fragmentation, were systematically investigated. It was observed that both the protonated molecular ions and major fragment ions from the active ingredients in tablets could be found in the full-scan mass spectra in positive ion mode, which were comparable to those obtained by a commercial LTQ-XL ion trap mass spectrometer. The structures of the ingredients could be elucidated in detail using the MEISD method, which promotes our understanding of the desorption/ionization processes in microwave plasma torch (MPT). Quantitative analysis of 10 tablets was achieved by full-scan MPTDI-TOF MS with low limit of detection (LOD, 0.763 mg/g), acceptable relative standard deviation (RSD < 7.33%, n =10), and 10 s for each tablet, showing promising applications in high throughput screening of counterfeit drugs.

**Electronic supplementary material:**

The online version of this article (doi:10.1007/s13361-017-1708-x) contains supplementary material, which is available to authorized users.

**Graphical Abstract:**
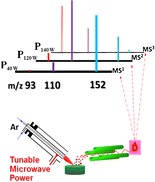
ᅟ

## Introduction

Identification and authentication of pharmaceuticals can be confidently achieved by mass spectrometry, which provides plentiful molecular information to probe the chemical nativity of all types of samples. Owing to the complexity of the tablet composition, targeted analytes must be separated from matrices before detection by mass spectrometry. The separation methods include HPLC [1], GC [2], CE [3], lab-on-chip system [4, 5], etc. These methods need laborious and time-consuming sample preparation. In 2004, Cooks et al. [6] demonstrated that analyte ions can be produced using desorption electrospray ionization (DESI) in an open-air environment without separating the matrix of real-world samples. From then on, more than 30 ionization methods have been reported for direct creation of analyte ions in ambient conditions such as direct analysis in real time (DART) [7], low temperature plasma (LTP) [8], extractive electrospray ionization (EESI) [9], and desorption atmospheric pressure chemical ionization (DAPCI) [10]. By using DESI and other ambient ionization techniques, throughput of mass spectrometry analysis was dramatically improved because no/minimal sample pretreatment was required [11–14].

To date, ambient ionization methods usually provide protonated/deprotonated analyte molecules for mass detection with a positive/negative ion detection mode, which simplifies the datum interpretation and improves the readability of the mass spectrum. Since the matrix is not removed in ambient mass spectrometry, multiple stage mass spectrometry experiments are normally required to obtain characteristic fragments of each analyte to exclude any potential false positive signals. Consequently, either a mass spectrometry instrument with extremely high resolution for exact mass measurement [15, 16] or tandem mass spectrometry capability (MS^n^, n ≥ 2) [17] is required to obtain confident results. At present, advanced instruments required to achieve either high mass resolution or multiple stage mass spectrometry experiments impose high cost, large size, and heavy weight on the mass spectrometers.

Apparently, these instruments are not suitable for in situ analysis, which limits the application of ambient mass spectrometry in many important fields such as chemical industry, process monitoring, drug discovery, and pharmaceutical screening. On the other hand, simple mass spectrometers have been constructed for onsite analysis [18–20]. More recently, miniature mass spectrometers installed with ambient ionization sources such as DESI [21, 22] and LTP [23] have been reported, showing increasing interest to develop inexpensive mass spectrometers with high analytical throughput for in situ applications.

Microwave plasma torch (MPT) has been used as the excitation source for atomic emission spectrophotometry (AES) for decades. Traditionally, the capability of MPT to produce ions has not been considered in AES. In fact, MPT combines many merits such as relatively high temperature, high electron density, and high positive charge density [24]. These make MPT a suitable ionization source for desorption ionization of solid samples. Recently, protonated analytes in liquid samples were also produced by MPT [25]. Theoretically, the high temperature of MPT allows the observation of ambient thermal dissociation of the analyte ions produced by MPT sources. In such a case, the total amount of the energy transferred to the analyte ion can be manipulated by changing the experimental conditions and, thus, the extent of fragmentation can be tuned without applying collision-induced dissociation (CID) experiments. Obviously, it is highly desirable to couple MPT with a simple mass spectrometer for rapid qualitative analysis of real-world samples. In this work, a novel, facile strategy is proposed to tune the mass spectral pattern by combining desorption and ionization process in a miniaturized MPT source. Because fast screening counterfeit drugs is of sustainable interest, MPTDI-MS was applied to rapid detection of ions of the active ingredients in drug preparations. The results showed installation of MPTDI-MS is cost-effective in potential screening of counterfeit drugs.

## Experimental

### Chemicals and Reagents

Ten kinds of tablets, including ribavirin (100 mg ribavirin/piece), azithromycin dispersible (250 mg azithromycin/piece), oxytetracycline (250 mg oxytetracycline/piece), metronidazole (200 mg metronidazole/piece), isoniazid (100 mg isoniazid/piece), compound paracetamol (126 mg paracetamol/piece), salbutamol sulfate (2 mg salbutamol/piece), acyclovir (100 mg acyclovir/piece), theophylline sustained-release (100 mg theophylline/piece), and amantadine hydrochloride (100 mg amantadine hydrochloride/piece) were purchased from local pharmacies. High-purity argon (99.999%) was obtained from Juyang Gas Co., Ltd. (Changchun, China). Ultrapure water (resistivity 18.2 MΩ cm^−1^) was produced by a Milli-Q device (Thermo Scientific, San Jose, CA, USA).

### Experimental Setup and Mass Analysis

**Figure 1:**
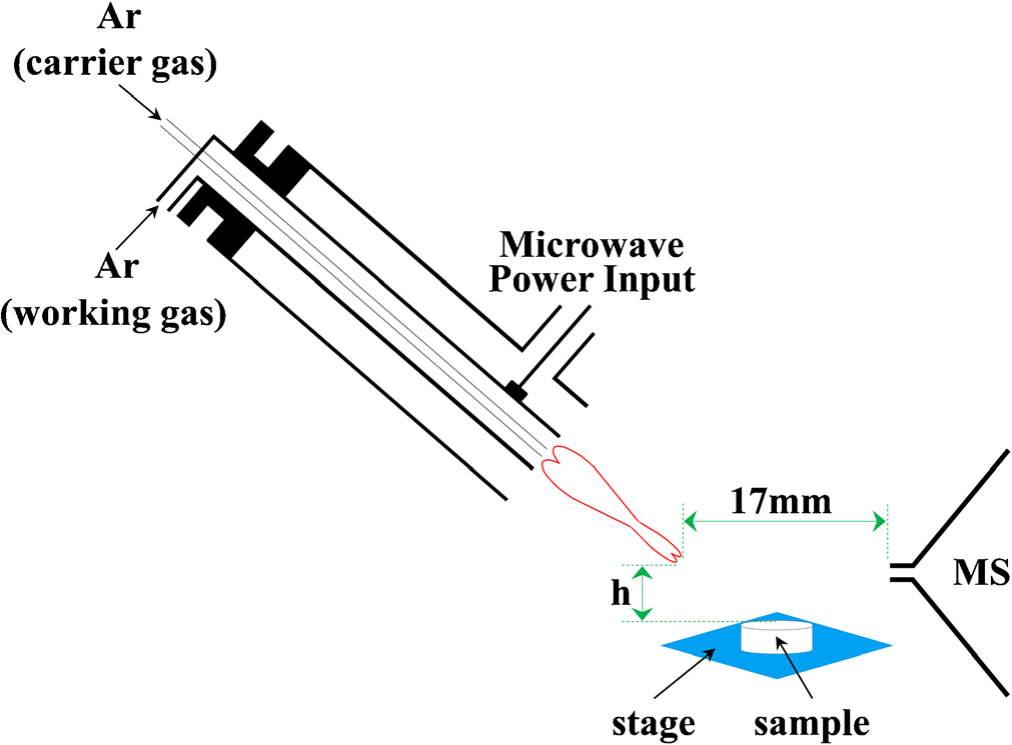
Schematic of MPT ion source coupled with a TOF MS. * Distance from MS inlet to plasma torch is fixed at 17 mm. The height of tablet surface to plasma torch is desorption distance h

Analysis was conducted with a time-of-flight mass spectrometer (TOF-MS 5000; Hexin Mass Spectrometry, Guangzhou, China) equipped with a MPT source with standard voltage, pressure, distance, and angling capability (Changchun Jilin University Little Swan Instrument, Jilin, China). The MPT ion source mainly consists of microwave plasma torch tube and microwave power source. Argon was used as plasma gas. The MPTDI-MS system is shown in Figure 1. LTQ-XL mass spectrometer (Thermo Scientific) was used for validation purpose in this study.

The instrument parameters were optimized. The configurations and operation of the MPT ion source were stated in detail in our previous work [26]. Microwave power was set to 50 watt, and the reflected power was reduced to almost 0 watt by adjusting the tuning piston of the microwave plasma torch tube. Stable argon plasma could be obtained when the flow rate of working gas (Ar) was 0.4 L/min and flow rate of carrier gas (Ar) was 1.2 L/min. In order to reduce the measurement error caused by the uneven distribution of active ingredient in tablets, the contact area of about 0.5 cm^2^ between plasma torch and the sample was controlled by adjusting the angle and distance of desorption (h).

The TOF-MS 5000 is a simple mass spectrometer with a relatively narrow mass range (*m/z* 50–800), a reduced size (70 cm length × 65 cm width × 75 cm height), and lighter weight (85 kg), and thus no tandem mass spectrometry experiment could be implemented in this compact TOF-MS instrument. Detailed description of the TOF-MS instrument was documented elsewhere [27]. The voltages were set as follows for this work: capillary voltage 140 V, focusing mirror voltage 135 V; positive pulse voltage 950 V, negative pulse voltage –950V, acceleration zone voltage −4100 V, grid voltage −90 V, reflex zone voltage 1340 V. No other optimization was made to the TOF-MS instrument. Thermo Finnigan LTQ-XL mass spectrometer is a widely available bench-top type commercial instrument, with advanced functions including multi-stage tandem mass spectrometry. The LTQ-XL instrument worked under the following conditions: the mass range was *m/z* 50–1000 with a positive ion detection mode, the temperature of the heated capillary was 150 °C, the lens voltage was 60 V. Other LTQ-XL parameters were automatically optimized by the system.

Pharmaceutical samples were placed on the sample holder without any pretreatment. The active ingredients of the tablets can be desorbed and ionized by microwave plasma. Then full-scan mass spectra of the samples were obtained using both MPT-TOF-MS instrument and LTQ-XL-MS instrument. For CID experiments, the precursor ions were isolated with a mass-to-charge window width of 1.6 Da, and then subjected to CID with collision energy of 15%–35%. Mass spectra were recorded with an average time of 30 s, and all backgrounds were subtracted.

## Results and Discussion

### Optimization of Desorption Condition

**Figure 2:**
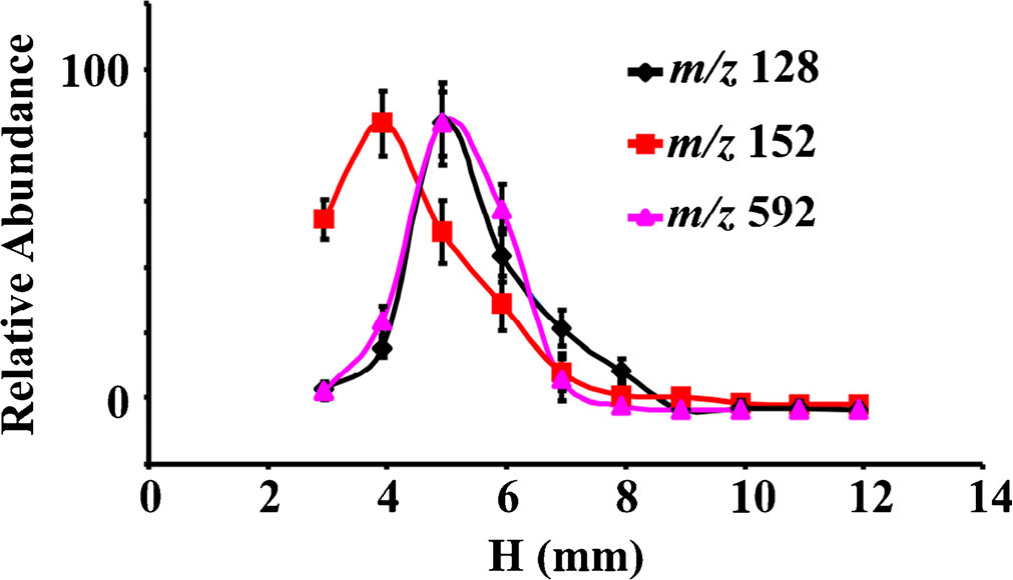
Effect of desorption distance (h) on abundance of major fragment ion of metronidazole (*m/z* 172→*m/z* 128), acyclovir (*m/z* 226→*m/z* 152), and azithromycin (*m/z* 750→*m/z* 592)

The distance from the apex of the torch to the tablet (h) and the time that the plasma worked on the sample (desorption time) were two of the most important parameters in the process of the MPTDI-MS experiment. The distance from the apex of the plasma to the ion entrance of the mass spectrometer was fixed at 17 mm (Figure 1). The influences of h and desorption time on signal intensities of analytes were studied. The representative plots obtained from metronidazole, acyclovir, and azithromycin are shown in Figure 2. The intensities of analyte ions achieved crest value when the distance was 4–5 mm. This trend could be explained from the perspective of energy and charge transfer. Because energy distribution of microwave plasma was variable in space, desorption and ionization capacities of the MPT were different in space. The abundance of fragment ions and ionization capacities increased when shortening desorption distance. However when the distance was too short (2–3 mm), the abundance of fragment ions decreased because the ionization capacity was too high and the fragment ions could be further cloven. Therefore, the MPT desorption distance of 5 mm was selected as the optimized parameter.

**Figure 3:**
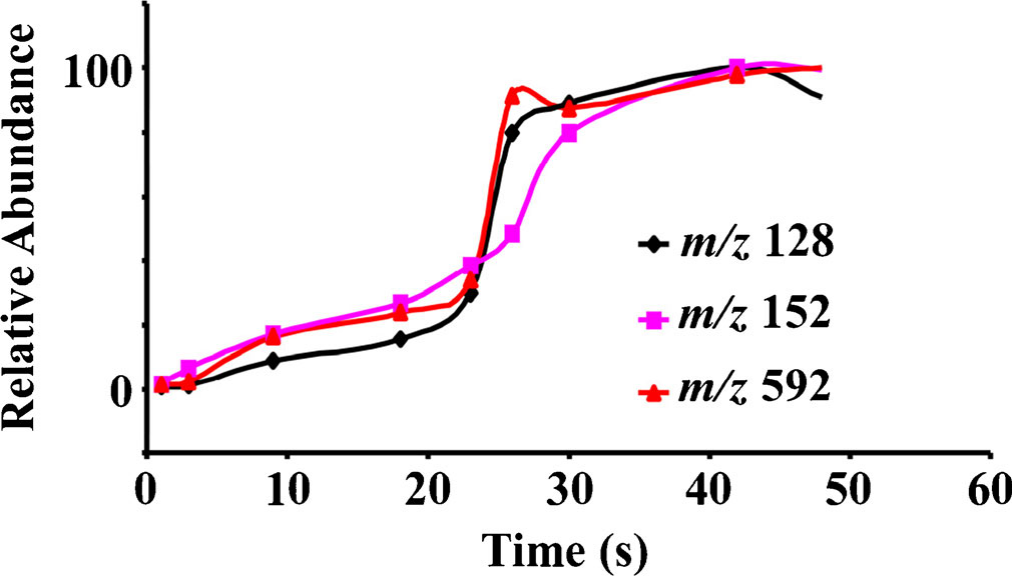
Desorption time (t) resulting the signal intensity variation of major fragment ion of metronidazole (*m/z* 128), acyclovir (*m/z* 152), and azithromycin (*m/z* 592)

In order to increase the ionization efficiency of MPTDI source, the relationship between desorption time and abundance of major fragment ions was studied; the curves of desorption time versus abundance for three typical pharmaceuticals are shown in Figure 3. This method could obtain the response signals of protonated molecular ions and fragment ions in less than 5 s. The results showed that efficiency of desorption and ionization increased with increasing desorption time, and significant changes of the signal intensities were observed in the range of 20–30 s desorption time, as depicted in Figure 3. Desorption times longer than 30 s did not lead to substantial increases in observed signals. With prolonged desorption time, not too much signal increase was observed. So the desorption time of 30 s was chosen as the optimal condition.

### Influence of Microwave Power on Ion Fragmentation

**Figure 4:**
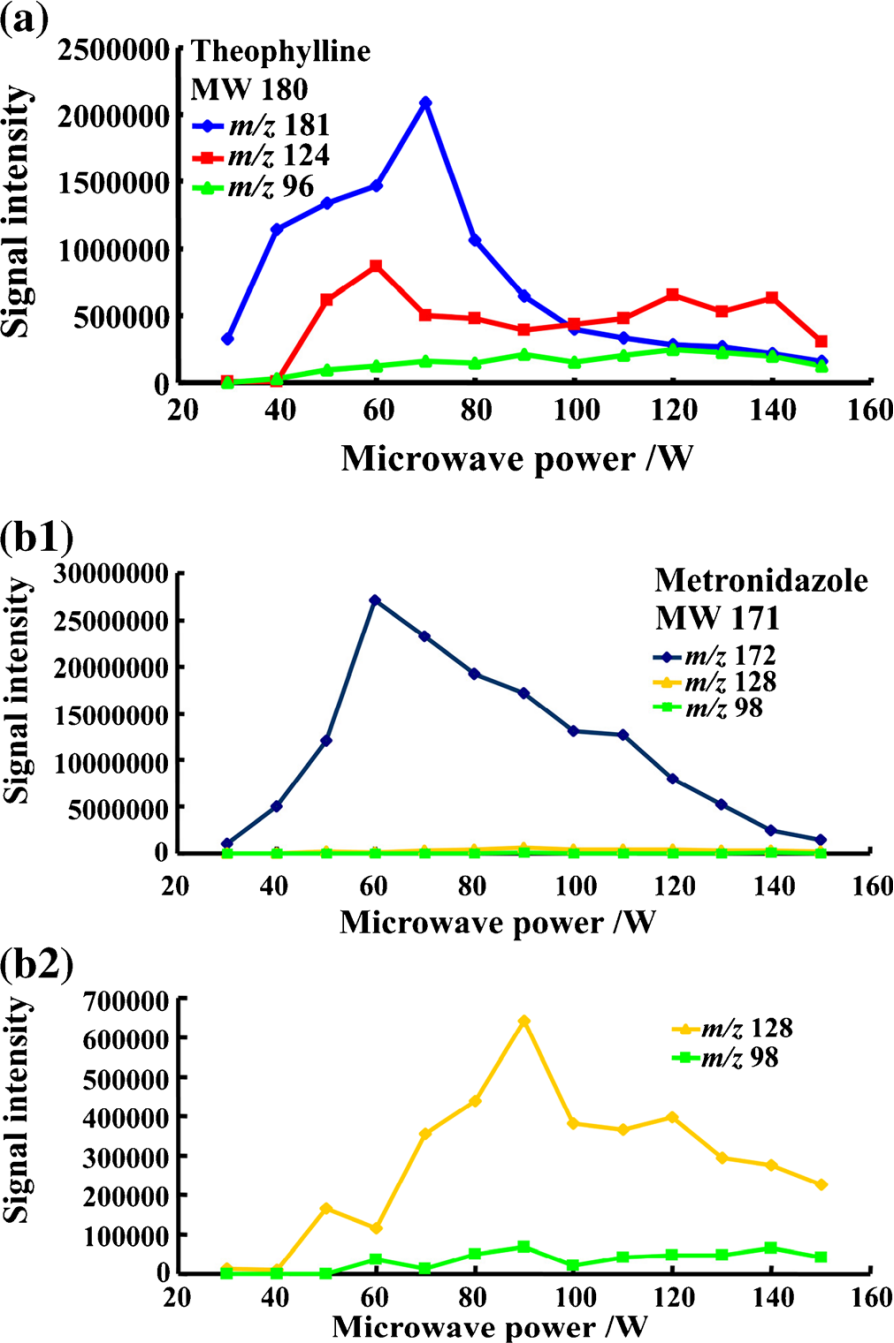
Influence of the microwave power of MPT ion source on signal intensities obtained for theophylline (**a**) and metronidazole, (**b**) in positive mode. *b1 is the signal intensity versus microwave curves for ions of metronidazole at *m/z* 172, 128, 98; b2 is the enlarged figure for ions at *m/z* 99 and 128. Instrument: MPT-TOF mass spectrometer

Microwave power is a key factor for ion fragmentation. The effect of microwave power on the signal intensities of main ions of analyte was investigated by using MPTDI-TOF MS. By raising the microwave power in increments of 10 watt from 30 to 150 watt, the protonated molecular ions with little fragmentation ions were mainly observed at low microwave power (≤40 W), as shown in the representative graph of theophylline (Figure 4a). The greatest signal intensities of the protonated species were observed when the microwave power reached to 50–70 watt for all 10 tablets. The highest signal intensity of protonated theophylline (*m/z* 181) with low signal intensity fragmentation ions (*m/z* 124, 96) were observed when the microwave power was at 70 watt (Figure 4a). In Figure 4b, the highest intensity of protonated metronidazole molecular ion at *m/z* 172 with its major fragment ions at *m/z* 128 and *m/z* 98 were observed at 60 watt. Higher microwave power (>60 watt) would induce MEISD and lead to high abundance of fragmentation ions, which corresponded to the MS^n^ (n =2, 3) fragmentation ions detected by MPTDI-LTQ MS in selective ion mode, indicating a valid MEISD. The fragmentation pathways that occurred in ion source of MPTDI-TOF MS were verified by tandem and multi-stage MPT-LTQ mass spectra of analytes (Supplementary Figure S-1c, j). The MS^2^ ion at *m/z* 124 was formed from the fragmentation of parent ion at *m/z* 181; the MS^3^ ion at *m/z* 96 was produced from the MS^2^ ion at *m/z* 124 by MPTDI-LTQ MS in selected ion mode. The MS^2^ ion at *m/z* 128 was produced from the fragmentation of parent ion at *m/z* 172; the MS^3^ ion at *m/z* 98 was formed from the MS^2^ ion at *m/z* 128.

In summary, high signal intensities of fragment ions, which are not typically observed in full scan MS with soft ambient ionization methods, were detected in our investigation. A key observation is that MPTDI-TOF MS is able to produce higher abundances of MS^n^ fragmentation ions when microwave power is set to >60 watt. The information of protonated molecular ions, molecular ions, and fragment ions could be obtained in the full-scan mass spectra of MPTDI-TOF under the conditions of 30 s of desorption time, 5 mm of desorption distance, and 70 watt of microwave power.

### Qualitative Analysis of Active Ingredients in Tablets

In this work, 10 kinds of commonly used pharmaceutical drugs were detected by MPTDI-TOF MS without any sample pretreatment. The CID data of drugs obtained by other researchers [28–42] are listed in Table 1.

**Table 1:**
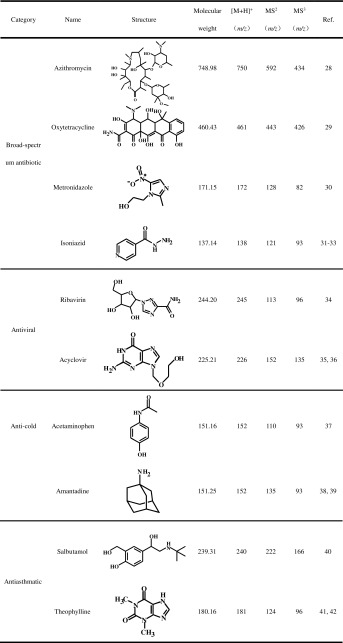
Related Information of Active Ingredients in Tablets

**Figure 5:**
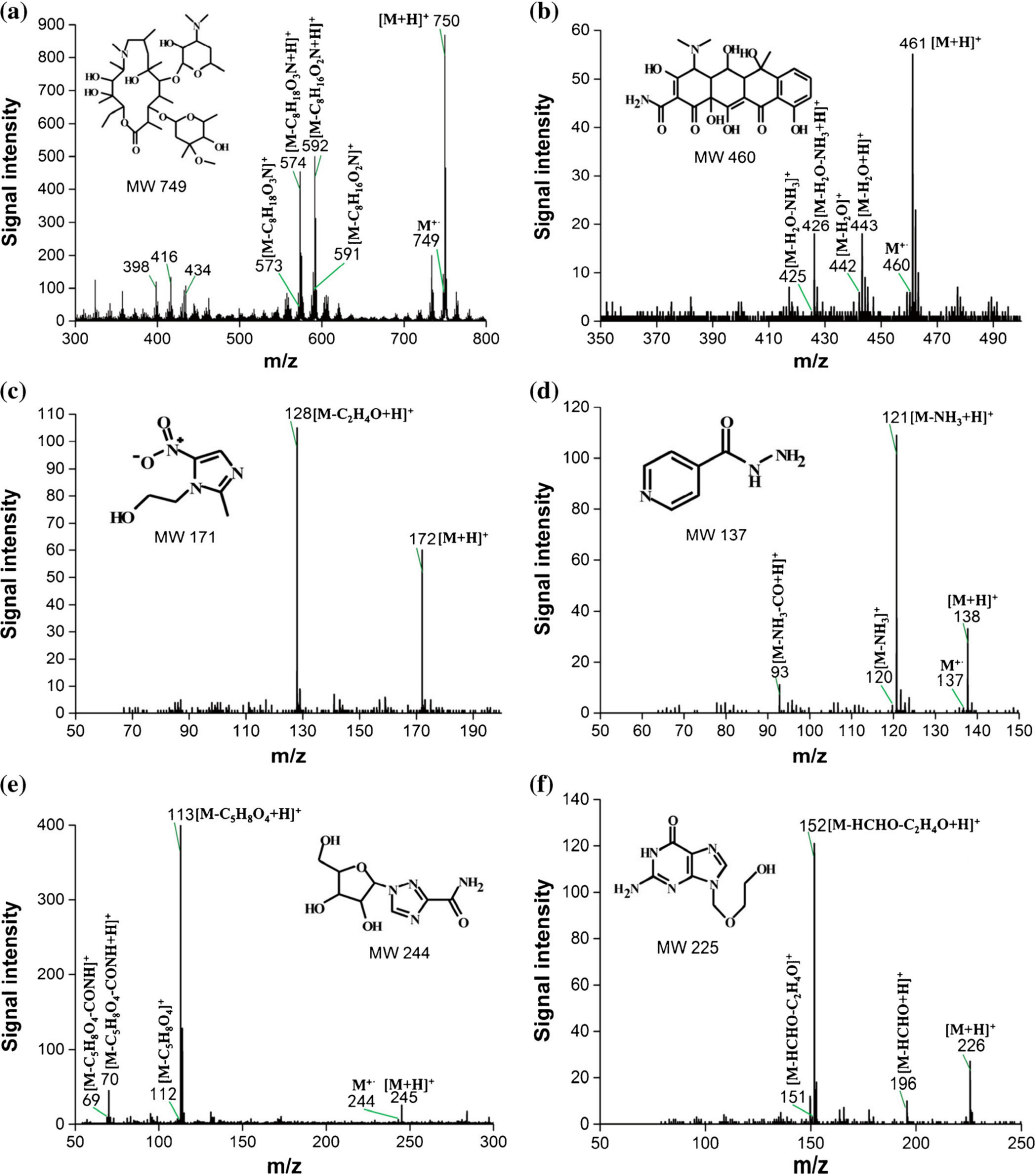
MPTDI-TOF full-scan mass spectra of (**a**) azithromycin; (**b**) oxytetracycline; (**c**) metronidazole; (**d**) isoniazid; (**e**) ribavirin; (**f**) acyclovir in positive mode

Azithromycin (MW 748.98) is a semi-synthetic and 15-membered ring macrolide antibiotic, and mainly used for treatment of respiratory tract infections caused by susceptible bacteria. The full-scan mass spectrum and fragmentation mechanism of azithromycin acquired by MPTDI-TOF MS are shown in Figure 5a and Supplementary Figure S-2a, respectively.

Main fragment ions of azithromycin at *m/z* 592,574,434,and 416 can be found in Figure 5a. In an effort to identify the analyte precisely, tandem mass spectrometric analysis was performed. The full-scan mass spectra data obtained by MPTDI-TOF MS were consistent with the MS^n^ data obtained by MPT-LTQ MS (Supplementary Figure S-1a).

Oxytetracycline (MW 460.43) belongs to an antibiotic tetracycline with broad-spectrum antimicrobial effect, and is one of the most commonly used pharmaceutical drugs. The main fragment ions of oxytetracycline, corresponding to peaks at *m/z* 443 and 426, can be found in the full-scan mass spectrum of MPTDI-TOF MS (Figure 5b), and similar result with MS^n^ data can be obtained by MPT-LTQ MS (Supplementary Figure S-1b). The protonated oxytetracycline contains multiple alcoholic hydroxyl groups and forms the ion at *m/z* 443 easily by losing a water molecule, and then losing an ammonia molecule to generate the ion at *m/z* 426. The possible fragment pathway of oxytetracycline at the MPT ionization source is shown in Supplementary Figure S-2b. It can be proven that microwave plasma thermal desorption and ionization at atmospheric pressure technology is expected to simplify qualitative analysis.

Metronidazole (MW 171.15) has a broad-spectrum antibacterial and antiprotozoal effect, and is mainly used for the prevention and treatment of infections caused by anaerobic bacteria. The mass spectra data obtained by MPTDI-TOF MS is shown in Figure 5c. It can be seen that there are two peaks at *m/z* 172 and 128. After the analysis of MS^n^ data obtained by MPT-LTQ MS (Supplementary Figure S-1c), it can be confirmed that the peaks at *m/z* 128 and 82 are the fragment ions of protonated metronidazole, and *m/z* 172 is the protonated molecular ion. The fragment ion at *m/z* 128 is likely formed by losing one molecule of vinyl alcohol from protonated metronidazole (Supplementary Figure S-2c). However, ion at *m/z* 82 is not found in the full-scan mass spectrum of MPTDI-TOF MS (Figure 5c), possibly because the bond cleavage to lose NO_2_ requires more energy.

Isoniazid (MW 137.14) is one of the widely used anti-tuberculosis drugs. Figure 5d shows the full-scan mass spectra of isoniazid obtained from MPTDI-TOF MS, protonated molecular ion at *m/z* 138, and main fragment ions at *m/z* 121, 93 can be found in the mass spectrum. This result is consistent with the MS^n^ result obtained by LTQ MS (Supplementary Figure S-1d). The possible fragment pathway was that protonated isoniazid occurred α-cleavage. The ion at *m/z* 121 corresponds to a loss of ammonia, ion at *m/z* 93 corresponds to a continuing loss of a carbon monoxide molecule. It can be proven that MEISD has both soft and hard ionization characteristics based on the analysis of the data obtained by MPTDI-TOF MS and MPT-LTQ MS.

Ribavirin (MW 244.20) is a potent broad-spectrum antiviral drugs and now widely used for the prevention and treatment of viral diseases. It can be seen that there are peaks at *m/z* 245 and 113 in the Figure 5e. It can be confirmed that these ions corresponded to protonated molecular ion and major fragment ion, respectively, based on the results in previous literature [34]. The fragment ion at *m/z* 113 for ribavirin analysis is due to the elimination of a stable 5-membered ring hydrofuran moiety. It was demonstrated that the MPT thermal desorption and ionization technology could achieve rapid and accurate qualitative analysis using only full-scan mass spectra data.

Similar with ribavirin, acyclovir (MW 225.21) is a kind of anti-viral drug, which is mainly used for herpes virus infection onset and recurrence. Both the protonated molecular ion *m/z* 226 and fragment ion *m/z* 152 of acyclovir can be observed in the full-scan mass spectrum obtained by the present simple TOF mass spectrometer, as shown in Figure 5f. Meanwhile, information of the fragment ion can be proven by the data from literatures [35, 36] and MS^n^ spectrum by MPT-LTQ MS (Supplementary Figure S-1f). The fragment pathway (Supplementary Figure S-2f) can be speculated that protonated acyclovir loses a methanal to form the ion at *m/z* 196. The ion at *m/z* 196 goes on to lose of oxirane (ethylene oxide) to produce the ion at *m/z* 152. According to the above results, the main fragment ion of acyclovir can be obtained without tandem mass spectrometry, and accurate qualitative analysis of acyclovir can be completed using only the data of full-scan mass spectrum by MPT ionization source coupled with a simple mass spectrometer.

Acetaminophen (MW 151.16), also named paracetamol, is a commonly used noninflammatory and antipyretic analgesic, and is the main component of many common cold medicines. The protonated molecular ion *m/z* 152 and fragment ions at *m/z* 110, and 93 of acetaminophen can be found in the full-scan mass spectrum of MPTDI-TOF MS (Supplementary Figure S-3g), which is consistent with the result obtained by MPT-LTQ MS. However, the peak at *m/z* 121 is observed due to hydrolysis reaction that occurred and acetic acid molecules that is produced. Then, acetic acid dimer molecules formed in MPT ion source, and strong peak at *m/z* 121 is observed. The ion at *m/z* 110 might be produced by losing the group of CH_2_CO from the protonated acetaminophen (Supplementary Figure S-2g). The protonated acetaminophen lost an acetamide molecule CH_3_CONH_2_ to form the fragment ion at *m/z* 93. Comparing the mass spectral data of Supplementary Figure S-3g with Supplementary Figure S-1g, it can be acknowledged that MPT ionization source coupled with a simple mass spectrometer can achieve the same effects as with a tandem mass spectrometer in the aspect of qualitative analysis.

Amantadine (MW 151.25) hydrochloride and acetaminophen are often added to cold medicine as main components. The protonated amantadine molecular ion and the fragment ions at *m/z* 152 and 135 can be observed in the full-scan mass spectrum (Supplementary Figure S-3h). The fragment ion at *m/z* 135 is consistent with that obtained by MPT-LTQ MS (Supplementary Figure S-1h). At the beginning of desorption and ionization, amantadine hydrochloride loses hydrochloric acid under the effect of microwave plasma. The fragment pathway of amantadine is relatively simple (Supplementary Figure S-2h). The peak at *m/z* 135 corresponds to the ion that formed by the loss of an ammonia molecule from protonated amantadine.

Salbutamol (MW 239.31) is a selective β2-receptor agonist, main effect for the treatment of asthmatic bronchitis, bronchial asthma, and emphysema-induced bronchospasm. The protonated molecular ion at *m/z* 240 and fragment ions at *m/z* 222, 166, and 148 can be found in the full-scan mass spectrum (Supplementary Figure S-3i). The ionic fragments of *m/z* 222, 166, and 148 are generated by the loss of H_2_O, [H_2_O+C(CH_3_)_2_CH_2_], [2H_2_O+C(CH_3_)_2_CH_2_]. According to the data of collision-induced dissociation of protonated salbutamol (Supplementary Figure S-1i), MPT ionization technology can help to obtain abundant, accurate information of protonated molecular ion and fragment ions of analytes without a tandem mass spectrometer.

Theophylline (MW 180.16) has direct effect on relaxation of airway smooth muscle, and is the drugs for bronchial asthma, asthmatic bronchitis, and other respiratory diseases. In the full-scan mass spectrum of theophylline obtained by MPTDI-TOF MS, the protonated molecular ion at *m/z* 181 and fragment ions at *m/z* 124, and 96 can be found (Supplementary Figure S-3j). The result is consistent with the MS^n^ data obtained by MPT-LTQ MS (Supplementary Figure S-1j) and reported in the literature. ISD fragmentation pathway of theophylline is proposed as depicted in Supplementary Figure S-2j. Six-membered ring of protonated theophylline can be opened under high energy, and thus loss of the CONCH_3_ group from protonated theophylline leads to the observation of the fragment ion at *m/z* 124, followed by the generation of the ion at *m/z* 96 by further loss of the CO molecule from the ion at *m/z* 124.

When analyzing active ingredients with the same molecular weight in solid pharmaceuticals such as acetaminophen and amantadine, DESI and DART ionization sources should be used in conjunction with tandem mass spectrometry for providing additional selectivity that helps to confirm the analytes. By changing the microwave power of MPT ionization source and selecting a proper desorption distance, ions information of tandem mass spectra of active ingredients were obtained in MPTDI-TOF MS without CID process, and the ingredients can be identified easily. In summary, a simple mass spectrometer coupled with MPT ionization source is a powerful tool for fast analysis of active ingredient in tablets.

### Probable Gas-Phase Reactions Caused by MPT

**Scheme 1:**
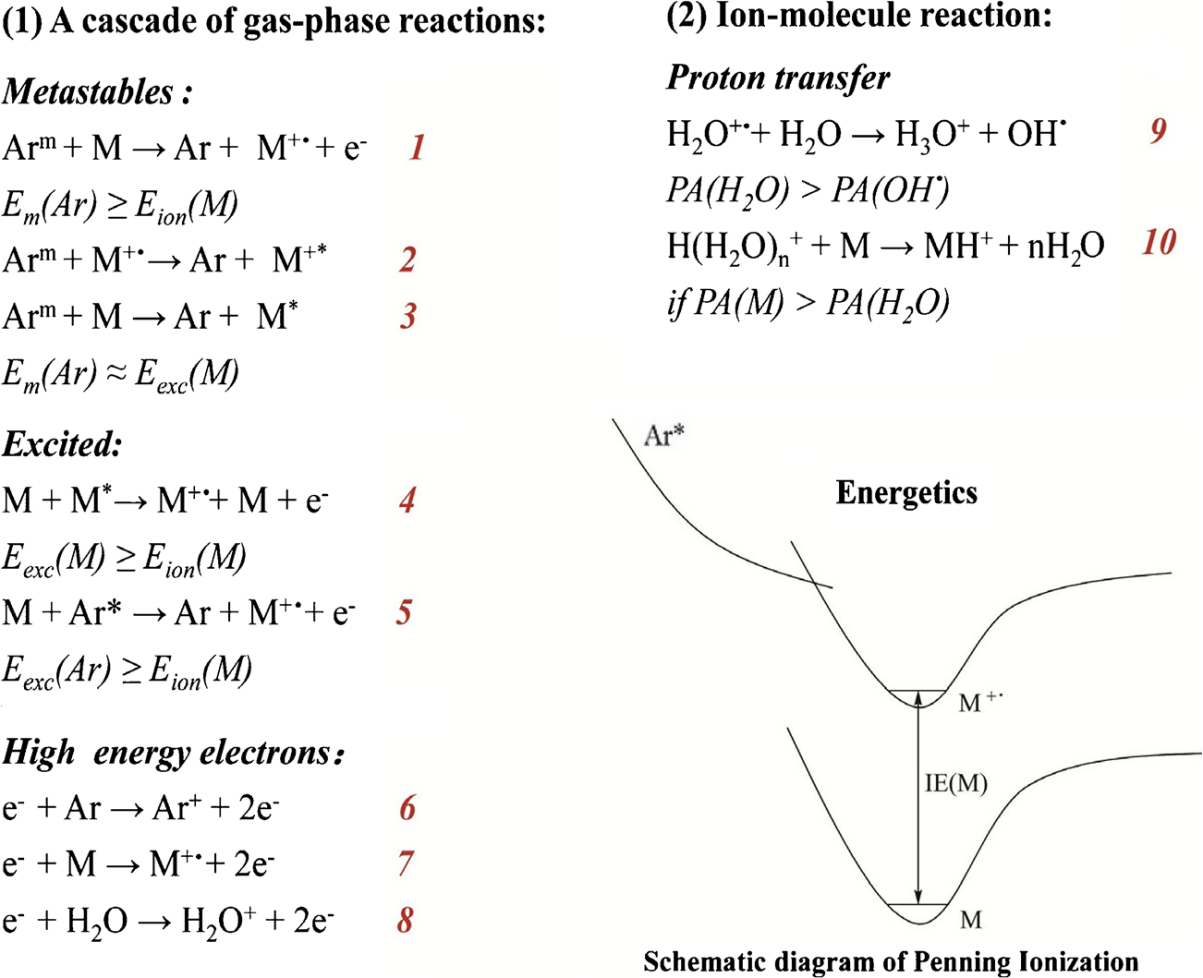
Probable Reaction Occurring in Ar-MPT. Ar_m_, the metastable states of argon; E_m_(Ar), the metastable energy of Ar; E_ion_(M), the ionization energy of M; E_exc_(M), the excitation energy of M; PA, proton affinity

Understanding how the ions work in MPTDI can be used to explore possible reactions in the gas phase and will be very helpful for researchers involved in the analysis or structural elucidation of compounds. During the Ar-MPTDI process, both protonated molecular ions and molecular ions of the analytes are observed (Figure 5). In Figure 5a, the base peak at *m/z* 750 corresponds to the protonated molecular ion of azithromycin. In addition, a relatively low-abundance molecular ion peak at *m/z* 749 is also detected. Similarly, both protonated molecular ions and molecular ions are observed in the full mass spectra of oxytetracycline (Figure 5b, *m/z* 461 and 460), isoniazid (Figure 5d, *m/z* 138 and 137), ribavirin (Figure 5e, *m/z* 245 and 244), acetaminophen (Supplementary Figure S-3g, *m/z* 152 and 151), salbutamol (Supplementary Figure S-3i, *m/z* 240 and 239), and theophylline (Supplementary Figure S-3j, *m/z* 181 and 180). It is speculated that these ions are generated by the reactions of Penning ionization (formation of molecular ions) and proton transfer (formation of protonated molecular ions). In the Ar-MPTDI process, Penning ionization ensues under an ultimate working condition of MPT, and molecular ions are generated by a cascade of gas-phase reactions. During the Penning ionization, the high densities of metastable argon (Ar_m_) atom [ionization energy (IE), 11.55 eV for the ^3^P_2_ state and 11.72 eV for the ^3^P_0_ state] [43] can ionize these investigated analyte molecules. The proposed reactions caused by metastable argon atoms are listed as reactions 1–3 in Scheme 1. They can take place since the energy of Ar_m_ is greater than the first or second ionization energy of analyte (M), E_m_(Ar) ≥ E_ion_(M). Reaction 1 is often followed by ion excitation (reaction 2). Reactions 2 and 3 involve direct excitation by metastable argon, and a more rigorous condition is required for the occurrence of reaction 2, E_m_(Ar) = E_exc_(M). Excited species with high excitation energies in the microwave plasma reacting with analyte molecules can generate molecular ions (reactions 4 and 5). Free electrons with high kinetic energy colliding inelastically with gas atoms in plasma and analyte molecules in the sample [44] is another reason for the generation of analyte molecular ions (reactions 6–8). These reactions are similar to those that happen in microwave-induced plasma (MIP) [45–48]. The protonated molecular ions are produced by proton transfer reaction, which is a dominant reaction in the MPTDI process. The proton affinity (PA) of water is 165 Kcal·mol^−1^, higher than that of hydroxyl radical (PA_OH•_ = 142 Kcal·mol^−1^) [43], so the proton transfer reaction between water and water molecular ion can occur, as shown in reaction 9. The protonated molecular ions [M + H]^+^ generated by proton transfer were observed because the analyte molecular ion M has a higher proton affinity (PAs of nitrogenous compounds are 200–240 kcal·mol^−1^ [49]) than the ionized water clusters (reaction 10). Although the molecular ions are also observed, the signal intensities are not high (Figure 5a, b, d, and e). They even disappear in MPTDI mass spectra of metronidazole (Figure 5c), acyclovir (Figure 5f), and amantadine (Supplementary Figure S-3h). Owing to the high PA of nitrogen atom and the relatively high PA of carbonyl oxygen, the added proton is initially located at them, and these analytes prefer to form protonated molecular ions with relatively high signal intensities during ionization. Thus, molecular ions are observed with low signal intensities or they disappear. The MS data in Figure 5 and Supplementary Figure S-3 confirms the reaction mechanism in MPTDI process proposed in Scheme 1.

Moreover, expected cleavage reactions that take place in the gas phase between MPT plasma and tablets produce abundant MS fragment ions, which provide rich structural information and would become a powerful tool to figure out the structure exactly by using a simple mass spectrometer. To clarify this phenomenon, we took azithromycin as an example. The ions at *m/z* 592 ([M-C_8_H_16_O_2_N + H]^+^), *m/z* 574 ([M-C_8_H_18_O_3_N + H]^+^), and ions at *m/z* 434 ([M-C_8_H_16_O_2_N-C_8_H_14_O_3_ + H]^+^), *m/z* 416 ([M-C_8_H_18_O_3_N-C_8_H_14_O_3_ + H]^+^) in MPTDI-TOF full-scan mass spectrum of azithromycin (Figure 5a) correspond to the MS^2^ ions and MS^3^ ions (Supplementary Figure S-1a) in tandem mode obtained by LTQ mass spectrometer, respectively.

Many applications using MPT ambient sources have been presented [24–26]. However, mechanisms for MPTDI have not yet been reported. Our investigation improves the understanding of the fundamental desorption and ionization processes in MPT, which will be helpful in the rapid and reliable identification of analytes in solid samples with complex matrices using MPTDI mass spectrometry.

### Quantitation of Active Ingredients in Tablet

**Figure 6:**
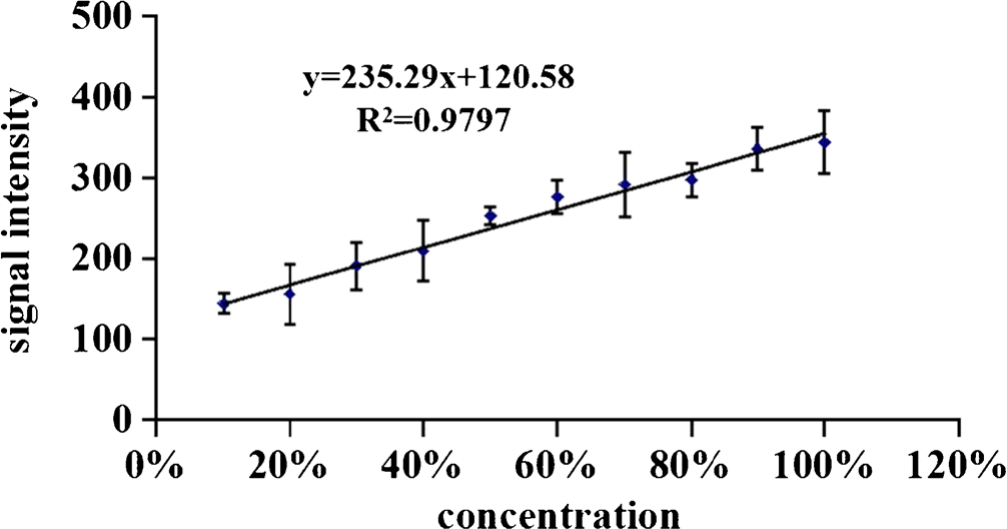
Calibration curve for acetaminophen. *The error bar in the curve is the standard deviation of net response signal

To examine the analytical performance of the present method, acetaminophen was used as the representative of the samples. Acetaminophen powder and corn starch were mixed (the ratio of acetaminophen powder in the mixture was 0%–100%) and then mixed powder was pressed into tablets using tableting machine. The re-pressed tablets containing acetaminophen were analyzed by the present method under the conditions of optimized desorption distance, time, and other parameters. The major fragment ion (*m/z* 110) of acetaminophen was selected as the quantitative ion and calibration curve was plotted using average value (n = 10) of net response signal of *m/z* 110 versus the concentration of acetaminophen powder in the mixture (Figure 6).

The results showed that the signal intensity and the concentration of acetaminophen displayed a good linear relationship in the 0%–100% range. The linear regression equation was I = 235.29 C + 120.58, and the correlation coefficient R = 0.9797. The real samples were analyzed and acetaminophen content was 60%. The relative standard deviation (RSD) was 7.33% (n = 10), and limit of detection (LOD, S/N = 3) of acetaminophen was 0.763 mg/g. The results showed that this method can meet the analysis of commercial tablets.

## Conclusions

In this paper, a new type ion source based on atmospheric pressure MPT coupled with a simple mass spectrometer is used to obtain protonated molecular ions, molecular ions, and fragment ions of the analytes by adjusting the desorption distance, desorption time, and microwave power. After the discussion of the structures and the MEISD mechanism of the obtained ions, the probable gas-phase reaction in MPT ion source is described. By comparison of the full-scan spectra obtained by the present method with the MS^n^ results obtained by MPT-LTQ MS, the accuracy of the present method is proven. The developed MPTDI-TOF-MS is expected to facilitate the qualitative and quantitative analysis of complex samples instead of using an expensive tandem mass spectrometer.

## Electronic supplementary material

Below is the link to the electronic supplementary material.

ESM 1 (DOC 5.11 mb)
