# Supplementary material for: Fast Determination of Ingredients in Solid Pharmaceuticals by Microwave-Enhanced In-Source Decay of Microwave Plasma Torch Mass Spectrometry
Source: J Am Soc Mass Spectrom. 2017 Jun 19;28(9):1947–57. doi: 10.1007/s13361-017-1708-x (PMC5556135; doi:10.1007/s13361-017-1708-x)
Supplement: Supplementary file 1 — (DOC 5.11 mb) [file 13361_2017_1708_MOESM1_ESM.doc]

**SUPPLEMENTARY MATERIAL**

**Fast Determination of Ingredients in Solid Pharmaceuticals by Microware-Enhanced In-Source Decay of Microwave Plasma Torch Mass Spectrometry**

Rui Su**^1,3￡^**, Xinchen Wang**^2￡^**, Changming Hou^1^, Meiling Yang**^2^**, Keke Huang**^1^***, Huanwen Chen**^2^,***

**^1^**Department of Chemistry, Jilin University, Changchun 130012, China

**^2^**Jiangxi Key Laboratory for Mass Spectrometry and Instrumentation, East China Institute of Technology, Nanchang, Jiangxi 330013, China

^3^Jilin Ginseng Academy, Changchun University of Chinese Medicine, Changchun，130117, China

^￡^There authors contributed equally to this work.

*Corresponding to: Dr. Huanwen Chen

Email: [chw8868@gmail.com](mailto:chw8868@gmail.com). Fax: (+86) 791-8389-6370. Tel.: (+86) 791-8389-6370.

This supplementary material is formed by Figures S1 to S3.


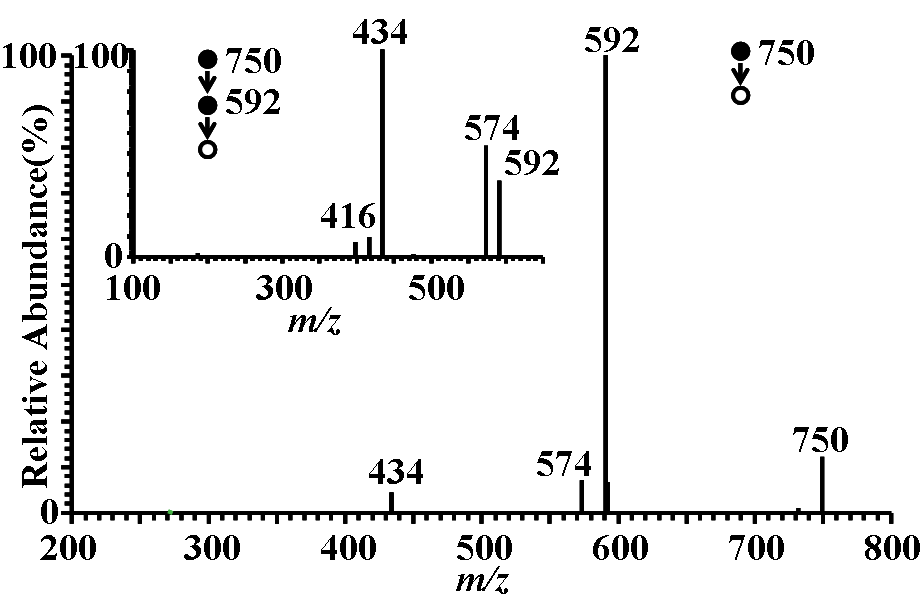

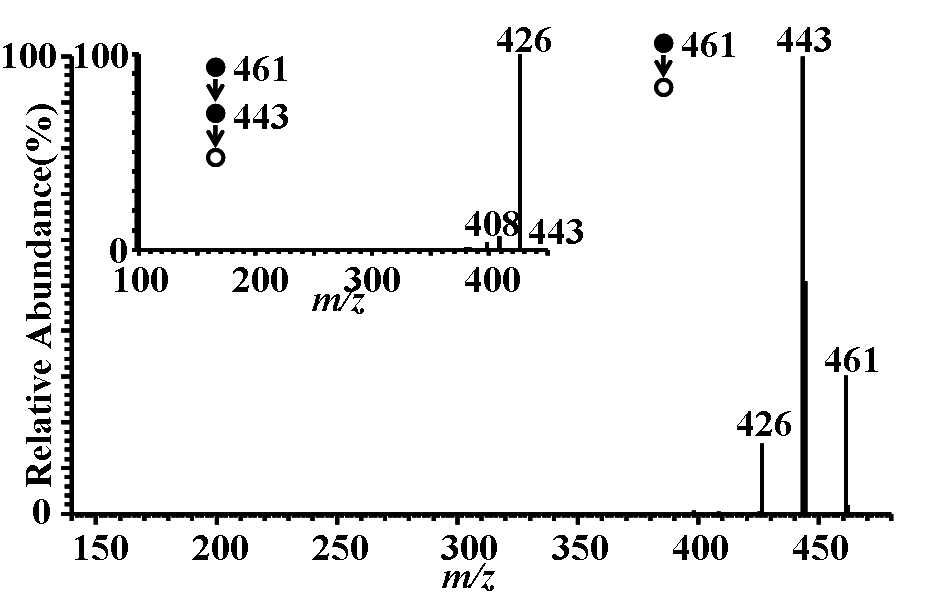


1. (b)


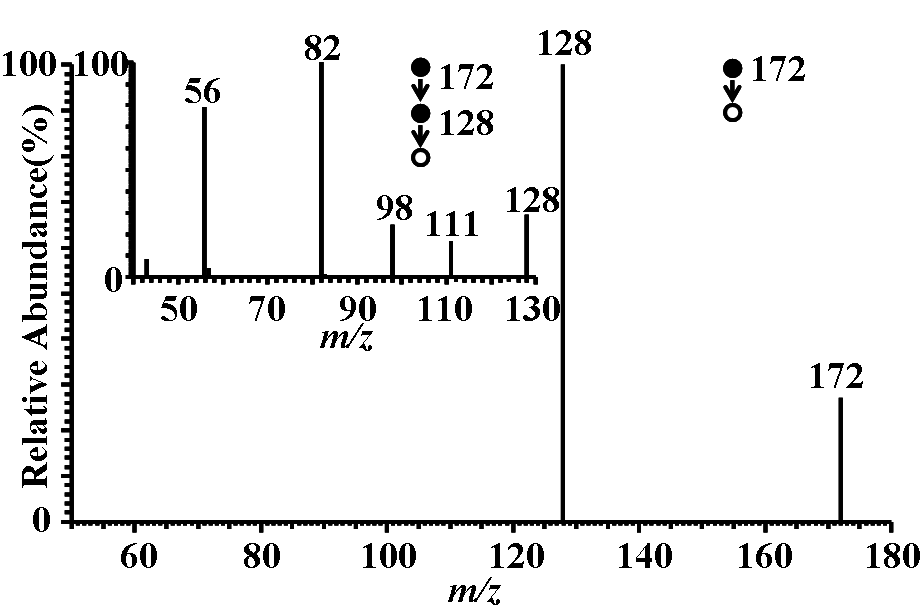

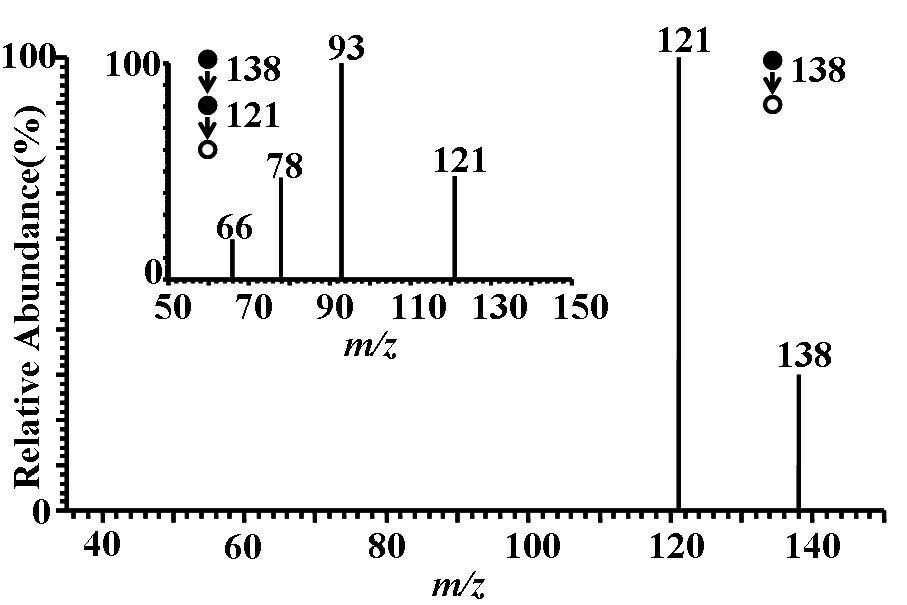


(c) (d)


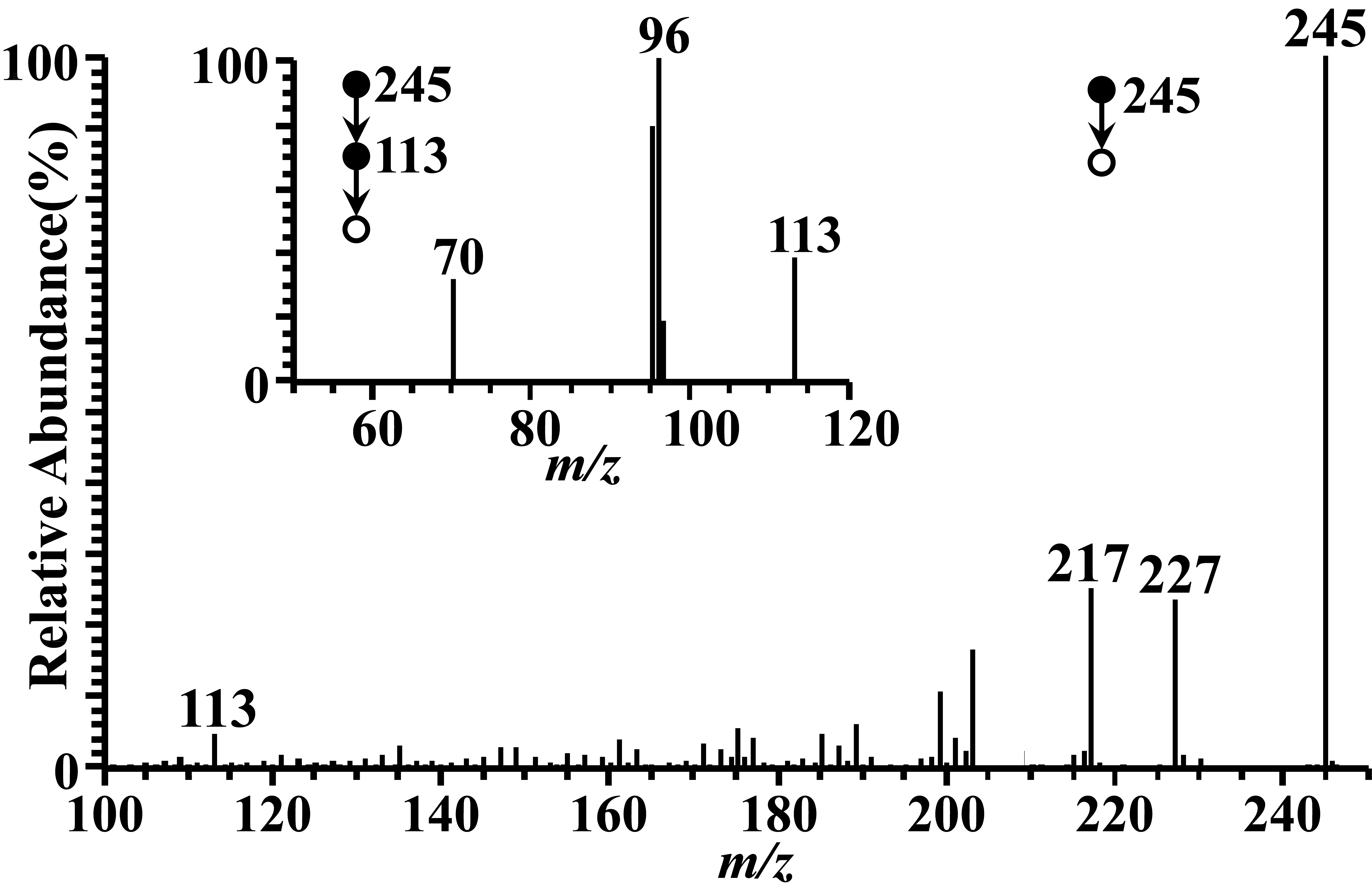


(e) (f)

(g) (h)

1. (j)

(i) (j)

Figure S-1. Tandem and multi-stage spectra of 10 ingredients from target tablets obtained using MPT-LTQ mass spectrometer. (a) azithromycin; (b) oxytetracycline; (c) metronidazole; (d) isoniazid; (e) ribavirin; (f) acyclovir; (g) acetaminophen; (h) amantadine ; (i) salbutamol; (j) theophylline.


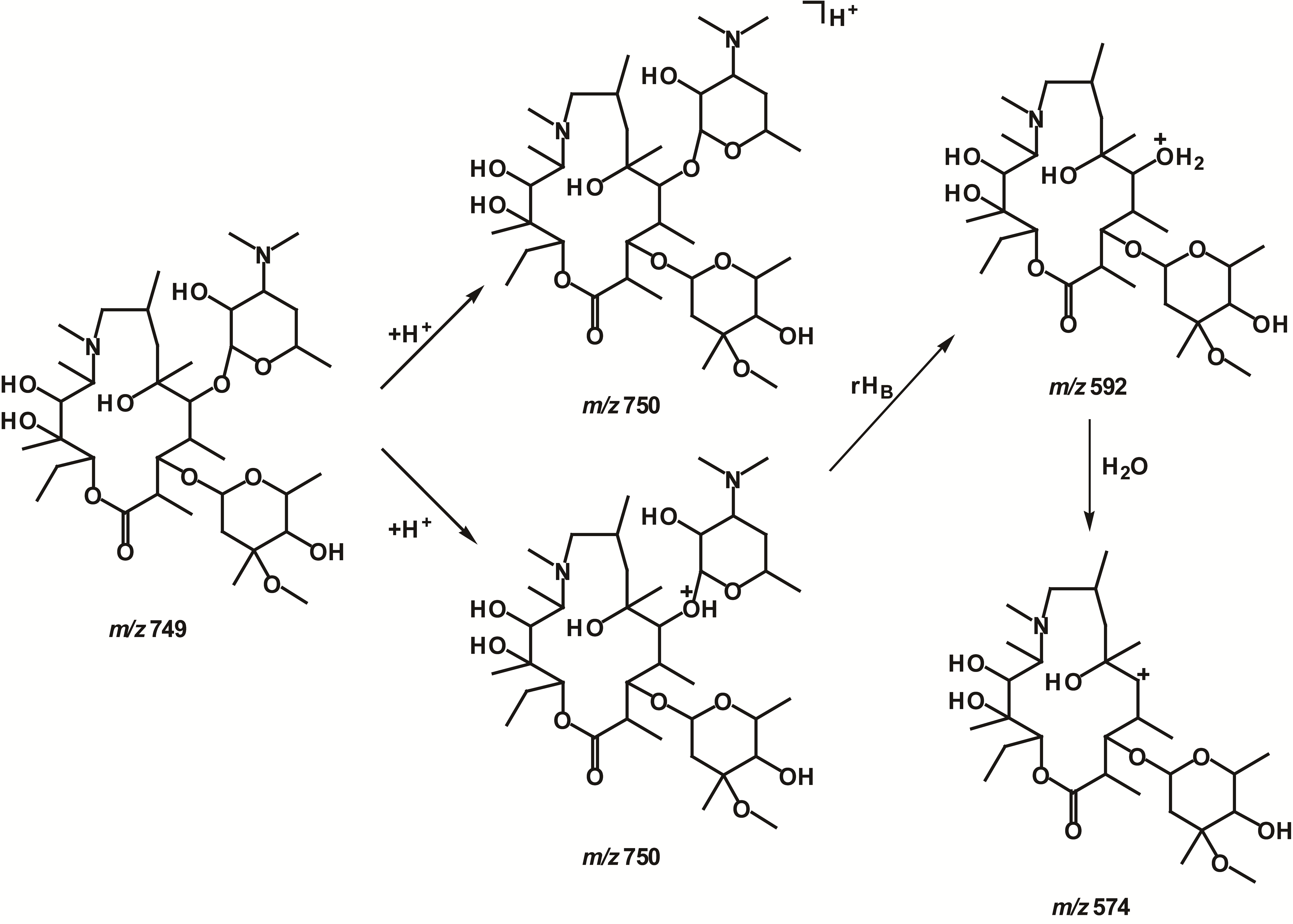


(a)


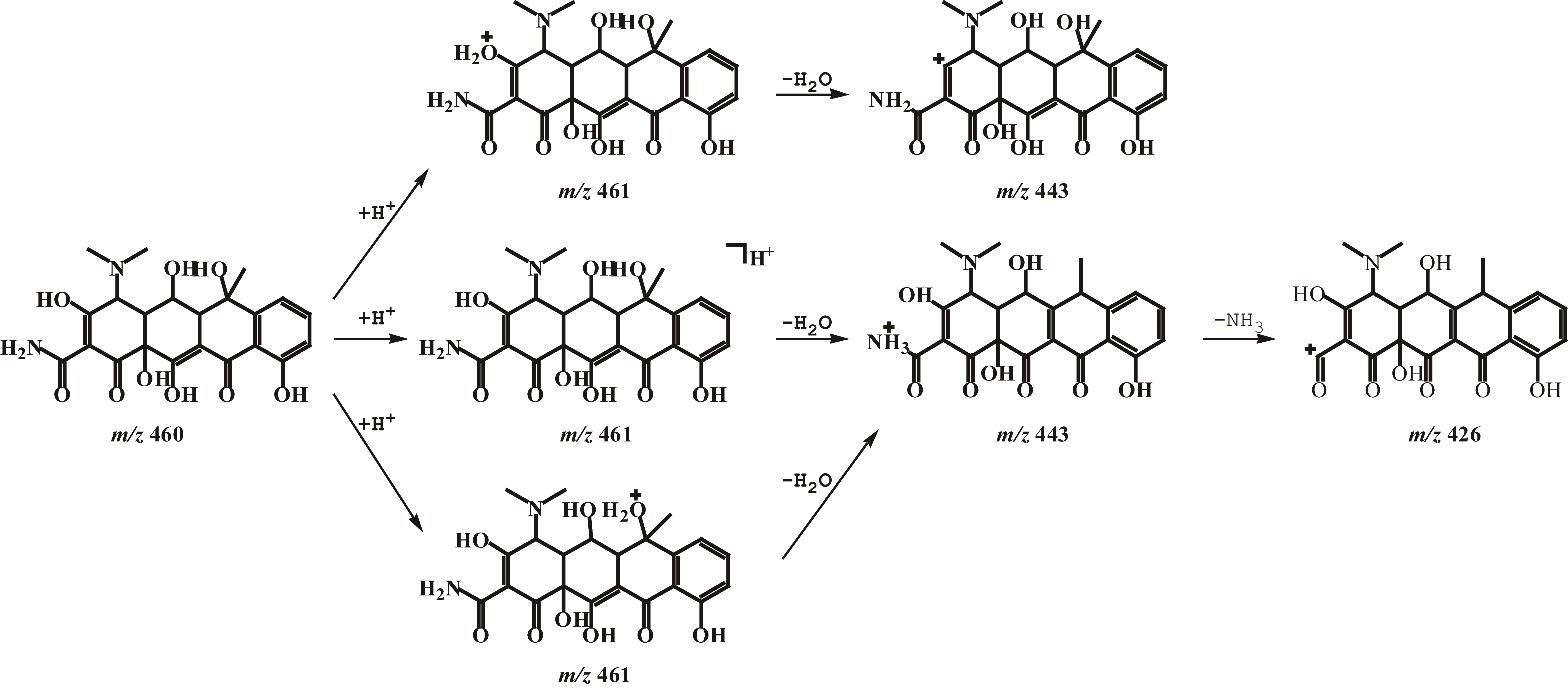


(b)


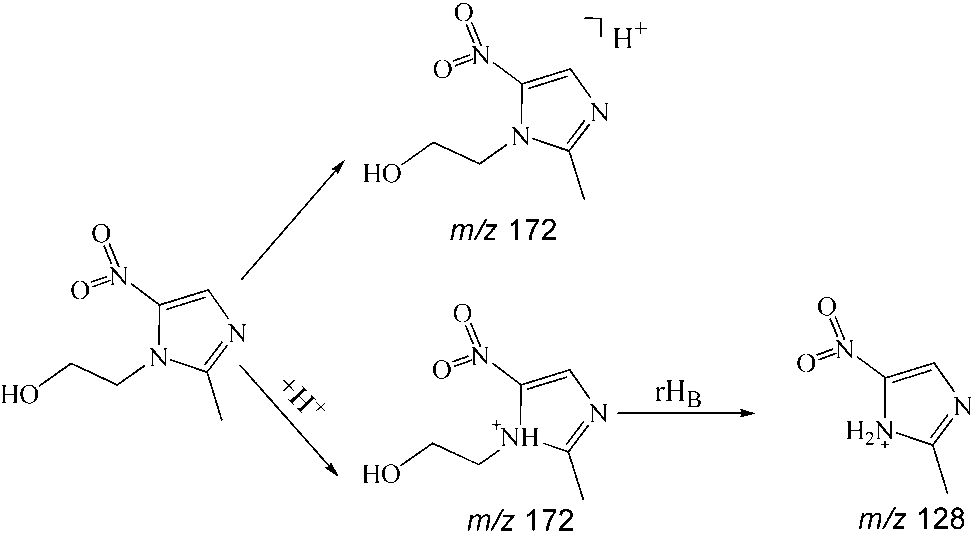


(c)

(d)


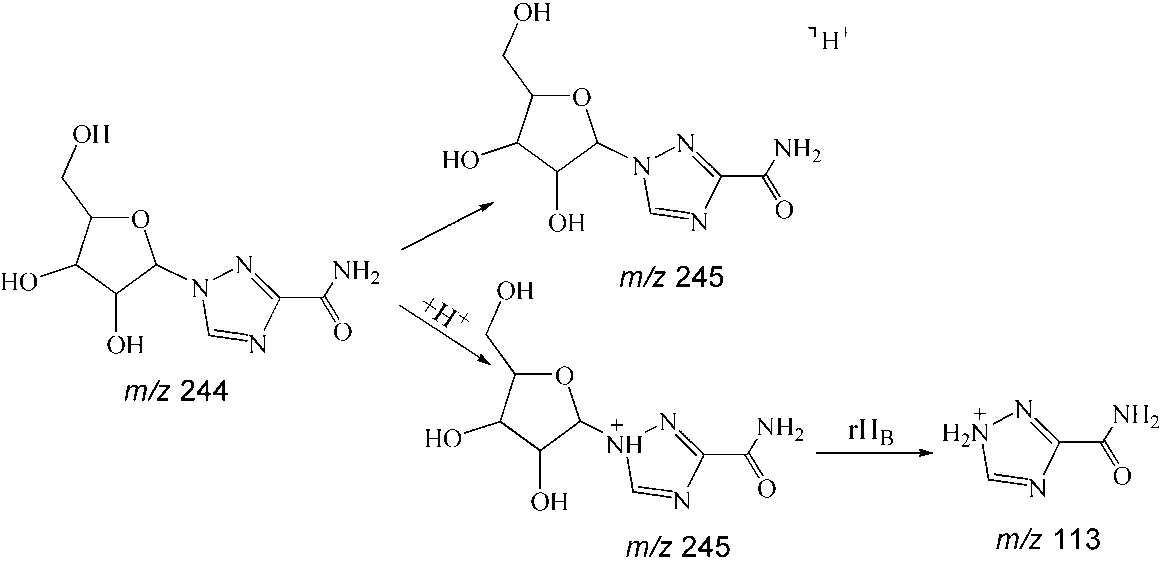


(e)


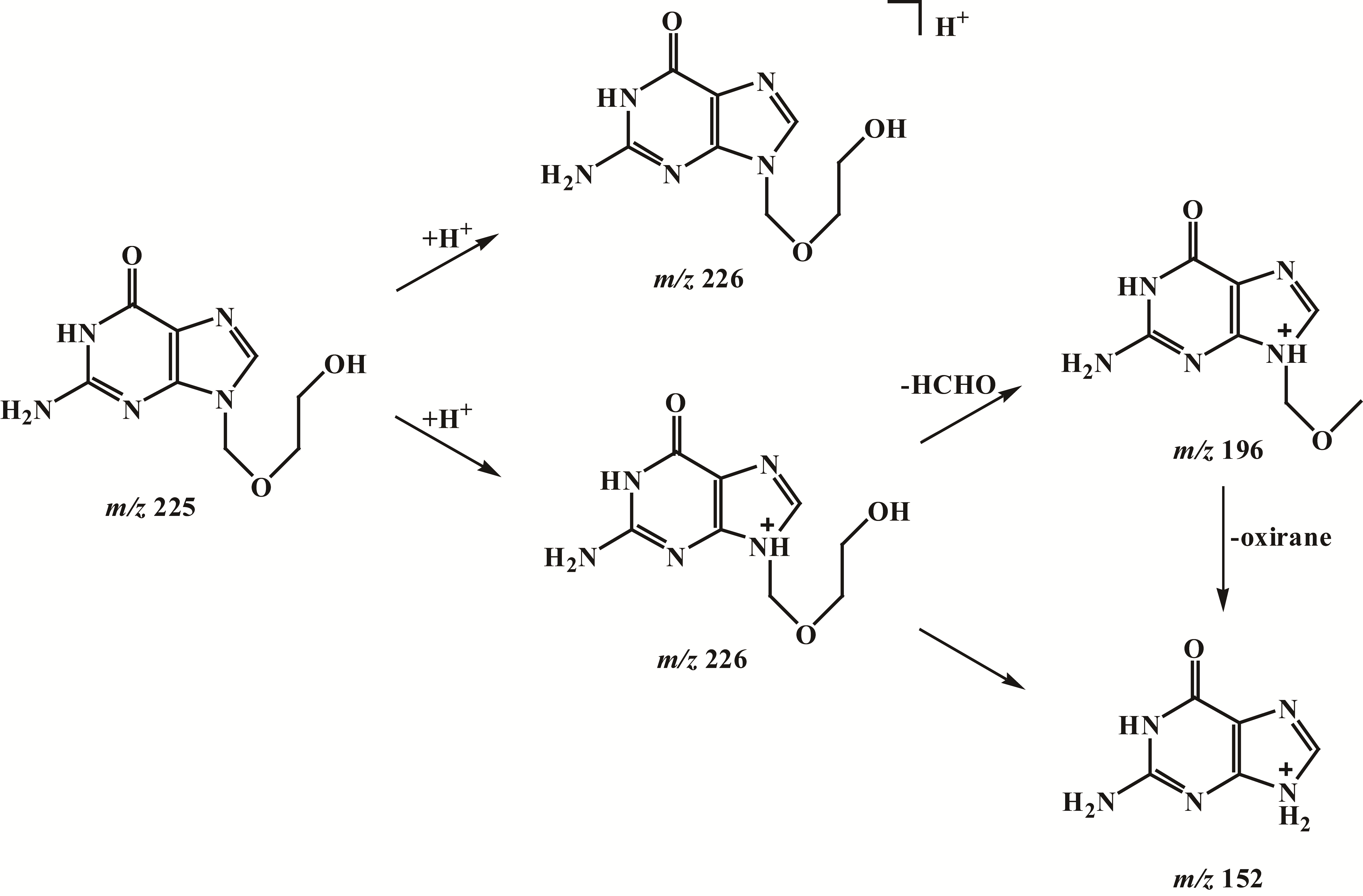


(f)


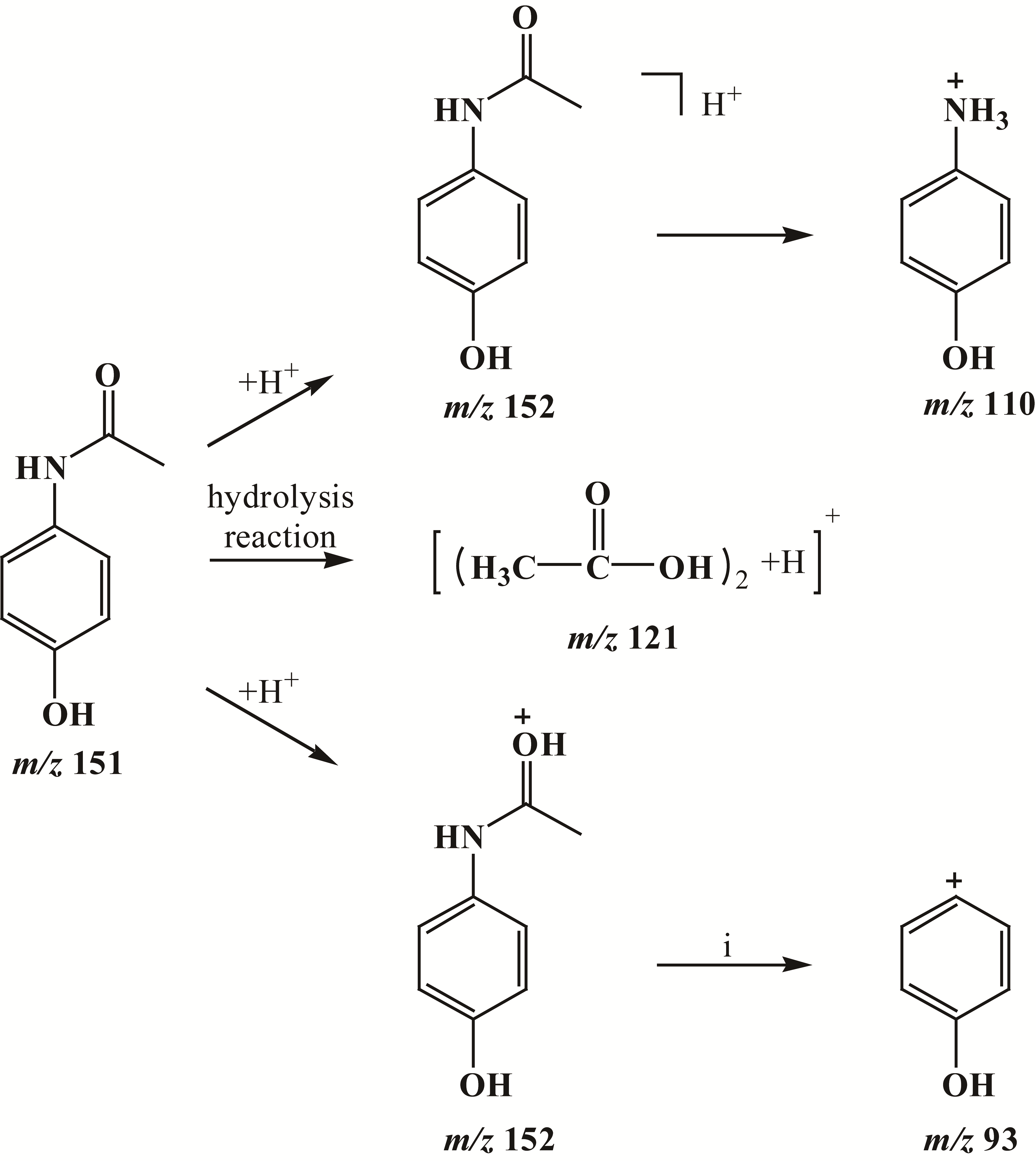


(g)


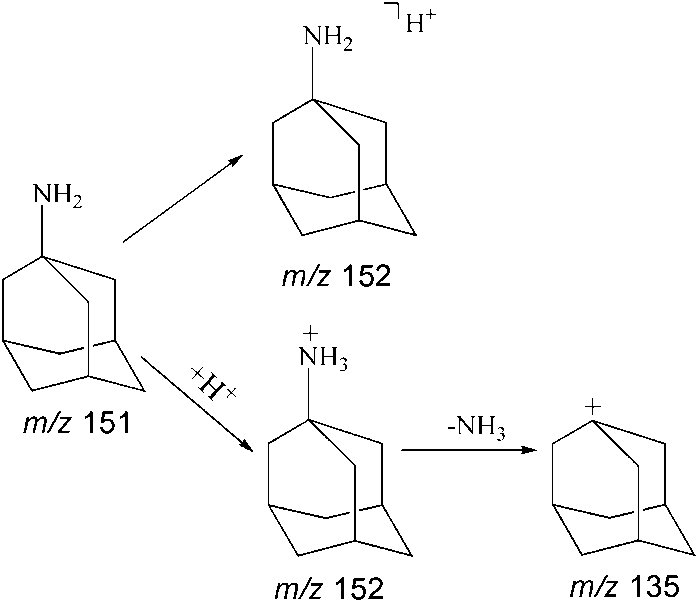


(h)


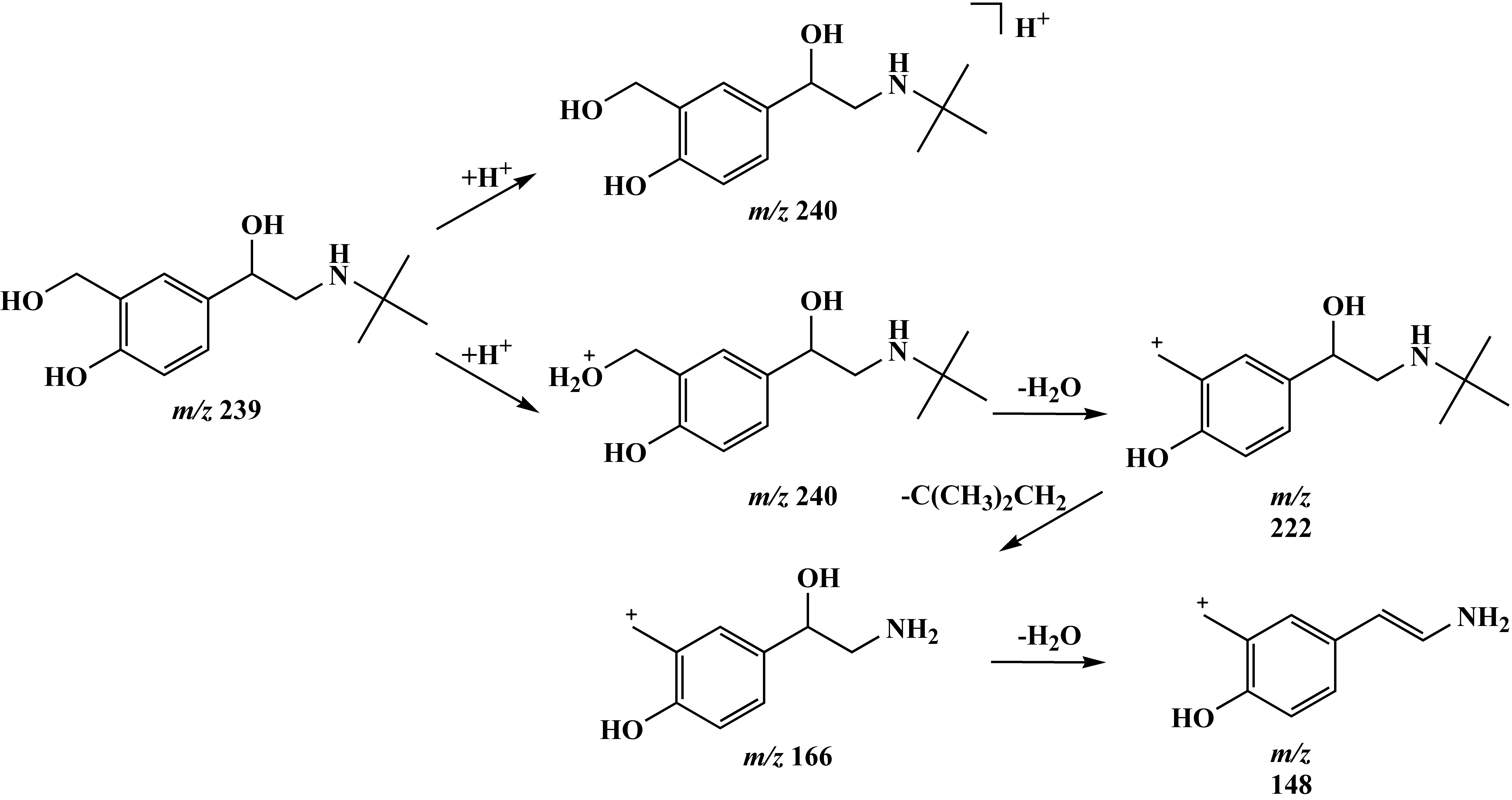


(i)


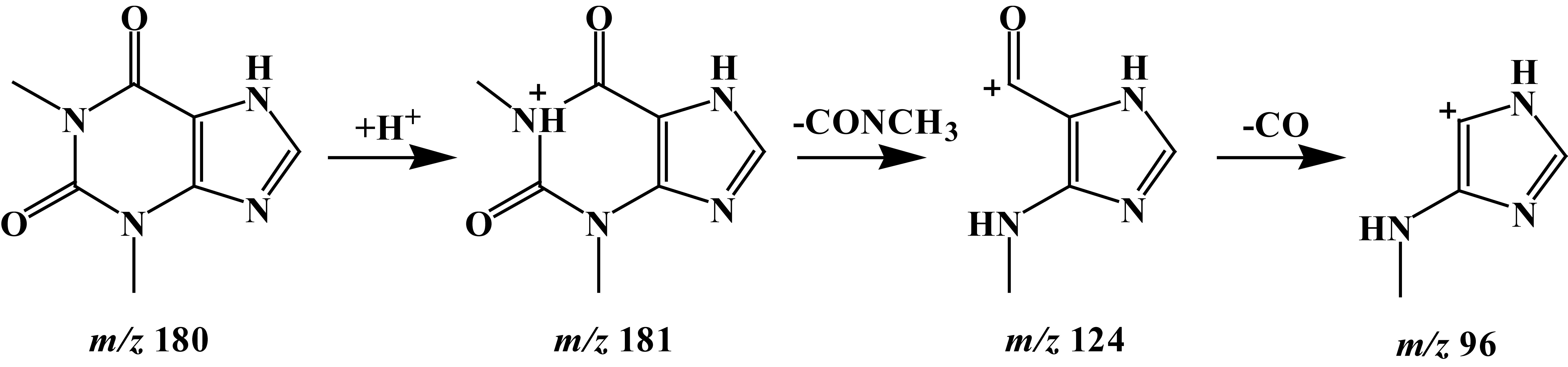


(j)

Figure S-2. MEISD mechanisms of 10 target ingredients in MPT ionization source. (a) azithromycin; (b) oxytetracycline; (c) metronidazole; (d) isoniazid; (e) ribavirin; (f) acyclovir; (g) acetaminophen; (h) amantadine ; (i) salbutamol; (j) theophylline.

* i, inductive cleavages; rH_B_, α, β-charrge site rearrangement.


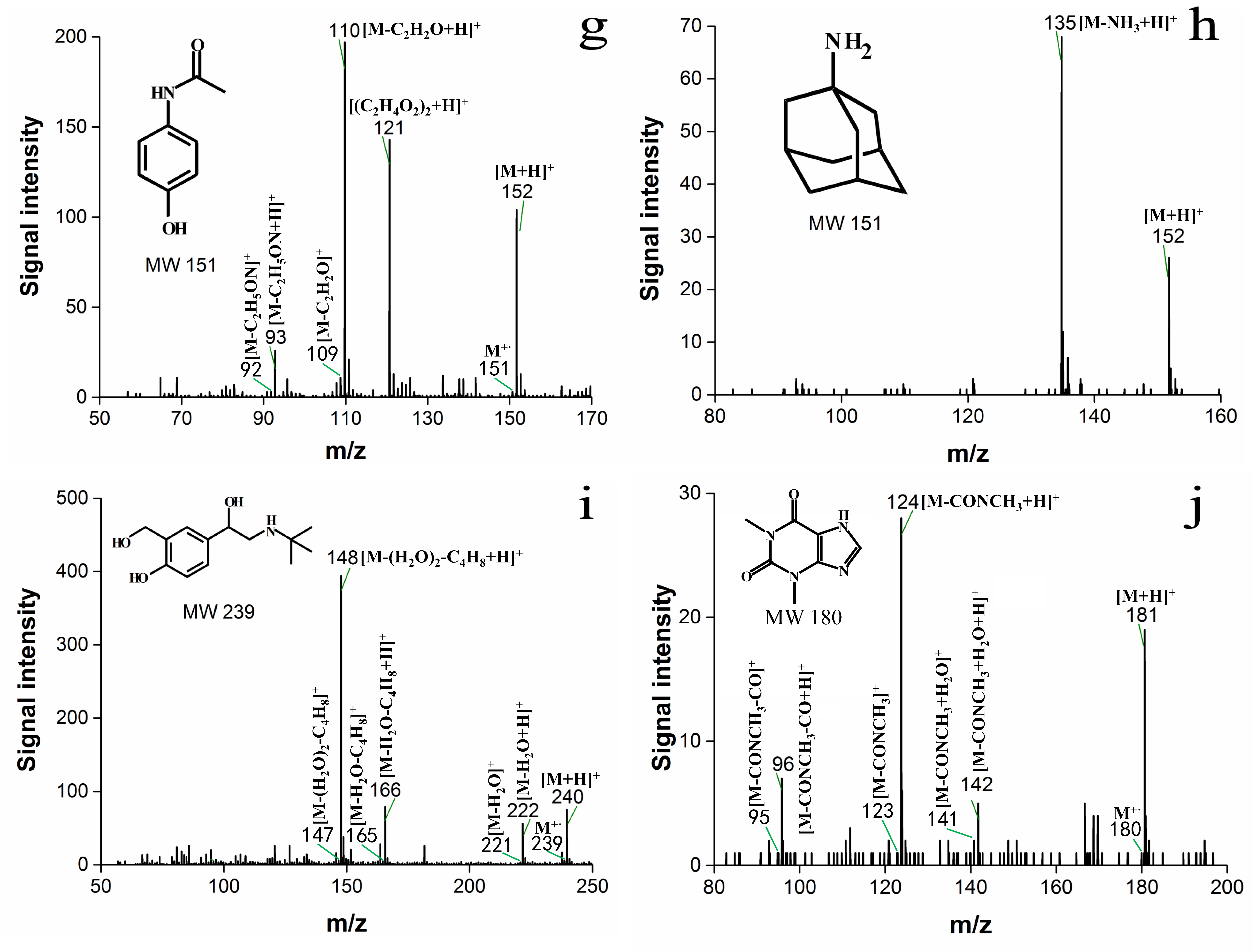


Figure S-3. MPTDI-TOF full-scan mass spectra of (g) acetaminophen; (h) amantadine; (i) salbutamol; (j) theophylline in positive ion mode.
